# The effect of preoperative psychological state on postoperative outcomes of anterior cervical approach in cervical spondylotic myelopathy: a retrospective case‒control study

**DOI:** 10.1186/s12891-025-09168-9

**Published:** 2025-10-14

**Authors:** Chunzeng Wang, Youdi Xue, Zhifa Lun, Ma Chao, Guangwang Liu, Xuan Yue, Zhaochuan Zhang

**Affiliations:** 1 Department of Spine Surgery, Southeast University Affiliated Xuzhou Central Hospital, Xuzhou, Jiangsu 221009 China; 2 Department of Spine Surgery, Xuzhou Central Hospital, Xuzhou, Jiangsu China; 3 Department of Neurology, Xuzhou Children’s Hospital, Xuzhou, Jiangsu China

**Keywords:** Cervical spondylotic myelopathy, Psychological state, Efficacy, Anterior cervical approach

## Abstract

**Objective:**

To explore the impact of the preoperative psychological state on postoperative clinical outcomes in patients with cervical spondylotic myelopathy(CSM) treated surgically by the anterior cervical approach.

**Methods:**

A retrospective study of 158 CSM patients undergoing anterior cervical surgery analyzed the impact of preoperative psychological state, measured by the preoperative mental state quality of life score (SF-12 MCS score). Patients were split into distress and non-distress groups for comparison. General data and clinical scores such as the Japanese Orthopaedic Association (JOA) score, neck disability index (NDI) score, visual analog scale (VAS) score, as well as patient satisfaction were assessed preoperatively and at final follow-up. Logistic analysis identified factors influencing psychological improvement in the distress group.

**Results:**

Among of 158 patients, 49 (31%) presented with preoperative psychological distress. This cohort experienced longer hospital stays and demonstrated poorer preoperative scores for NDI, physical health (PCS), and mental health (MCS). Both groups achieved significant and equivalent improvement in physical outcomes (JOA, VAS, NDI). Although the distress group showed greater improvement in MCS scores, their postoperative satisfaction rate (81.6%) was numerically lower than that of the non-distressed group (89.9%), a difference that was not statistically significant. A higher final mental health score was associated with a better preoperative MCS, a longer follow-up period, and male sex.

**Conclusions:**

Patients with CSM exhibit a high incidence of psychological distress. Anterior cervical surgery effectively improves both physical symptoms and mental health outcomes in these patients. Key factors associated with poorer mental health recovery include a low preoperative MCS score, shorter follow-up duration, and female sex. Importantly, preoperative psychological distress does not negatively affect the improvement in neurological function or overall postoperative satisfaction.

## Introduction

Cervical Spondylotic Myelopathy (CSM) is a chronic, progressive condition caused by age-related degeneration of the cervical spine. It is characterized by intervertebral disc degeneration and herniation, which, along with posterior osteophytes, compress the spinal cord and its blood supply (vasculature), leading to a distinct set of neurological symptoms. Current treatment guidelines recommend surgical intervention as the primary approach for definitively diagnosed CSM [1, 2]. Surgical strategies include anterior and posterior approaches, the former including cervical discectomy and fusion (ACDF) or cervical corpectomy and fusion (ACCF), and the latter including laminoplasty or posterior laminectomy and fusion. For degenerative cervical disorders with three segments or fewer, anterior approach may be a good choice [2].

Notably, patients with degenerative spinal disorders frequently exhibit comorbid psychological conditions, with more than one-third of surgically treated individuals reporting anxiety, depression, or other psychological abnormalities [3]. Previous studies have shown that preoperative psychological distress in patients with cervical radiculopathy or those undergoing posterior cervical surgery adversely affects postoperative functional recovery and satisfaction [4–6]. However, it remains unclear whether preoperative psychological distress similarly influences clinical outcomes in CSM patients following anterior cervical surgery.

This study aimed to retrospectively analyze clinical data from CSM patients who underwent anterior cervical surgery to determine the prevalence of psychological distress in this population, compare postoperative outcomes between patients with and without preoperative psychological distress, and identify factors influencing psychological improvement in affected individuals. These findings may provide valuable insights for optimizing clinical management.

## Methods

### Study design

The inclusion criteria were as follows: (1) a definitive diagnosis of CSM, confirmed by spinal cord compression as evidenced through magnetic resonance imaging (MRI) and computed tomography (CT). (2) First surgical intervention for CSM, which may include either ACDF or ACCF. (3) Availability of comprehensive follow-up data for a minimum duration of 24 months. (4) No history of psychiatric disorders or related therapeutic interventions.

The exclusion criteria were as follows: (1) the presence of cervical radiculopathy, congenital cervical deformities, ankylosing spondylitis, or spinal cord compression due to cervical fractures. (2) Treatment of CSM involving posterior cervical surgical techniques. (3) Coexisting neurogenic or myopathic conditions. (4) Incidents of cervical infections or the presence of primary or metastatic spinal tumors. (5) A history of previous spinal surgical procedures.

We conducted a retrospective analysis of 158 patients from the cervical spondylosis registry database and medical records at Xuzhou Central Hospital. These patients met the established inclusion and exclusion criteria between July 2019 and May 2022. The cohort consisted of 86 men and 52 women, with ages ranging from 33 to 84 years (mean age: 57.59 ± 11.64). The BMI of the participants varied from 21.16 to 31.65 kg/m² (mean BMI: 25.32 ± 1.98).

This study received ethical approval from the Ethics Committee of Xuzhou Central Hospital (Approval No. XZXY-LJ-20200107-013), and a waiver for informed consent was granted owing to the retrospective nature of the investigation.

### Data collection

Demographic and clinical data, including sex, age, surgical level, BMI, disease duration, total hospital stay, and preoperative hospitalization period, were collected.

### Outcome measures

#### Disease-related pain assessment

Neck pain severity was assessed preoperatively and at the final follow-up using the visual analog scale (VAS). The percentage improvement in the VAS score was calculated as follows: The VAS improvement percentage is determined by subtracting the final follow-up score from the preoperative score, dividing by the preoperative score, and multiplying by 100.

#### Quality of life assessment (SF-12 V2)

Health-related quality of life was evaluated using the 12-Item Short Form Survey Version 2 (SF-12 V2), which comprises two domains: the physical component score (PCS) and the mental component score (MCS) (range: 0–100; higher scores indicate better status). The MCS, a validated tool for screening psychological distress [7–9], was used to categorize patients into two groups: the psychological distress group (MCS < 50) and the nondistress group (MCS ≥ 50).

The percentage improvement in the MCS was defined as follows: The MCS improvement percentage is derived by subtracting the preoperative score from the final follow-up score, dividing by the preoperative score, and multiplying by 100.

#### Functional outcomes

Japanese Orthopaedic Association (JOA) score: Cervical myelopathy severity was graded pre- and postoperatively. The percentage improvement in the JOA score was calculated as follows: The JOA improvement percentage is determined by subtracting the preoperative score from the postoperative score, dividing by the maximum possible improvement (17 - preoperative score), and multiplying by 100.

The outcomes were stratified as follows: excellent (> 60% improvement), moderate (25–60%), or poor (< 25%).

Neck Disability Index (NDI): The percentage improvement in functional disability was quantified using the following formula: The NDI improvement percentage is derived by subtracting the final follow-up score from the preoperative score, dividing by the preoperative score, and multiplying by 100.

#### Postoperative satisfaction

Patient satisfaction was assessed at the final follow-up using a 5-point Likert scale: very dissatisfied, dissatisfied, neutral, satisfied, and very satisfied responses were dichotomized for analysis as Satisfied: “satisfied” or “very satisfied”. Dissatisfied: “neutral”, “dissatisfied” or “very dissatisfied”.

### Statistical analysis

Statistical analysis was conducted using SPSS 25.0 software (IBM, USA). Quantitative data that conformed to a normal distribution are expressed as the mean ± standard deviation, and comparisons between groups were performed using independent sample t tests. Categorical data are represented as frequencies (rates), and comparisons between groups were conducted using the χ² test. A P value of less than 0.05 was considered statistically significant.

## Results

All patients completed the follow-up. Among the 158 patients, 49 (31.01%) with CSM experienced preoperative psychological distress, as indicated by an MCS of less than 50. In contrast, 109 patients (68.99%) did not experience psychological distress (MCS ≥ 50). Those in the distress group had significantly longer preoperative and total hospital stays than did those in the nondistress group (both *P* < 0.001). Additionally, a greater percentage of patients in the distress group underwent multilevel surgical procedures (involving two or more segments: 79.6% vs. 56.9%), although this difference did not reach statistical significance (χ² = 1.260, *P* = 0.020). No other significant differences were noted in the baseline demographics (*P* > 0.05) as shown in Table 1.

**Table 1:** Demographic and preoperative characteristics of the psychological distress and nondistress groups

Variable	Psychological Distress Cohort (*n*=49)	Nondistress Cohort (*n*=109)	Statistical Value	*P* Value
Sex (*n*, %)			χ²=0.851	0.356
Male	24 (49.0%)	62 (56.9%)		
Female	25 (51.0%)	47 (43.1%)		
BMI (mean, kg/m²)	25.22 ± 2.04	25.35 ± 1.96	t=−0.394	0.694
Age (years)	56.37 ± 12.17	58.15 ± 11.41	t=−0.867	0.376
Disease duration (months)	9.18 ± 1.99	9.00 ± 1.79	t=0.571	0.568
Surgical approach (*n*, %)			χ²=1.260	0.262
ACDF	29 (59.2%)	54 (49.5%)		
ACCF	20 (40.8%)	55 (50.5%)		
Surgical levels (*n*, %)			χ²=7.806	0.020
1	10 (20.4%)	47 (43.1%)		
2	17 (34.7%)	30 (27.5%)		
3	22 (44.9%)	32 (29.4%)		
Preoperative hospitalization (days)	3.24 ± 1.64	2.23 ± 0.74	t=5.374	<0.001
Total hospitalization (days)	10.71 ± 3.00	8.22 ± 1.47	t=7.025	<0.001
Follow-up duration (months)	27.34 ± 1.76	27.16 ± 1.89	t=0.569	0.570

Data are presented as the means ± SDs or %, bold *P* values indicate statistical significance (*P* < 0.05)

*ACDF* Anterior cervical discectomy and fusion, *ACCF* Anterior cervical corpectomy and fusion, *BMI* Body mass index

### Clinical outcomes

#### Disease-specific scores

There were no significant differences in the preoperative or final follow-up JOA or NDI scores between the two groups (all *P* > 0.05). Both groups, however, showed significant improvements in JOA and NDI scores at the final follow-up compared with baseline (both *P* < 0.001) as shown in Table 2.

**Table 2:** Comparison of clinical outcomes between groups

Clinical Score	Psychological Distress Group (*n* = 49)	Nondistress Group (*n* = 109)	Statistical Value	*P* Value
JOA score				
Preoperative	10.73 ± 1.48	10.81 ± 1.46	t=−0.28	0.773
Final follow-up	14.63 ± 1.01*	14.47 ± 1.09*	t = 0.84	0.399
Improvement (%)	60.81 ± 17.95	57.44 ± 19.73	t = 1.01	0.309
VAS score				
Preoperative	5.78 ± 0.90	5.73 ± 0.76	t = 0.28	0.779
Final follow-up	1.96 ± 0.64*	2.05 ± 0.76*	t=−0.74	0.462
Improvement (%)	0.64 ± 0.14	0.63 ± 0.15	t = 0.21	0.837
NDI score (%)				
Preoperative	48.82 ± 6.27	36.32 ± 3.59	t = 13.02	< 0.001
Final follow-up	22.43 ± 2.62*	22.43 ± 2.67*	t = 0.01	0.992
Improvement (%)	36.72 ± 10.18	37.74 ± 9.09	t=−0.63	0.529
PCS score				
Preoperative	37.88 ± 5.16	35.69 ± 7.01	t = 2.19	0.031
Final follow-up	44.82 ± 3.64*	45.51 ± 4.07*	t=−0.99	0.322
Improvement (%)	20.14 ± 17.14	32.43 ± 29.55	t=−2.71	0.007
MCS score				
Preoperative	39.73 ± 3.94	52.65 ± 2.16	t=−21.57	< 0.001
Final follow-up	49.18 ± 3.56*	52.57 ± 3.06	t=−6.13	< 0.001
Improvement (%)	24.82 ± 13.67	3.91 ± 6.82	t = 12.83	< 0.001
Satisfaction (*n*, %)			χ²=2.09	0.148
Dissatisfied	9 (18.4%)	11 (10.1%)		
Satisfied	40 (81.6%)	98 (89.9%)		

*P* < 0.05 indicates statistical significance, * denotes significant improvement compared with the preoperative values

*JOA* Japanese Orthopaedic Association, *VAS* Visual analog scale, *NDI* Neck disability index, *PCS* Physical component score, *MCS* Mental component score

#### Quality of life and satisfaction

- Physical Component Score (PCS): No significant differences were observed in the preoperative or final PCS between the groups (*P* = 0.322 and *P* = 0.031, respectively). Nonetheless, the improvement rate in the PCS was significantly greater in the distress group (*P* = 0.007). Both groups demonstrated significant enhancements in PCSs compared with their baseline measurements (*P* < 0.05).
- Mental Component Score (MCS): Compared with the nondistress group, the distress group showed significant postoperative improvement in MCS (*P* < 0.05) and had notably lower preoperative and final MCSs (both *P* < 0.001). Furthermore, the degree of improvement in the MCS was significantly greater in the distress group (*P* < 0.001).
- Satisfaction: Satisfaction proportions at the final follow-up were 81.6% for the distress group and 89.9% for the nondistress group, with no significant difference between the groups (χ² = 2.09, *P* = 0.148)(Table 2).

#### Factors influencing postoperative MCS in the distress group

Among the 49 patients who experienced preoperative psychological distress, 25 (51.02%) achieved an MCS of 50 or greater at follow-up (indicating improvement), whereas 24 (48.98%) remained below 50 (indicating persistent distress). Logistic regression analysis identified several significant predictors of the final MCS:

- A 1-point increase in the preoperative MCS was associated with a 0.78-point increase in the final MCS (*P* < 0.05).
- Each additional month of follow-up corresponded to a 0.15-point increase in the MCS (*P* < 0.05).
- Female sex was associated with a 2.45-point reduction in the final MCS (*P* < 0.05).

Age, BMI, and surgical approach did not significantly affect MCS outcomes (*P* > 0.05)(Table 3).

**Table 3:** Univariate and multivariate regression analyses of factors associated with an MCS ≥ 50 at the final follow-up in the psychological distress group

**Variable**	**Univariate Analysis**				**Multivariate Analysis**			
	**β**	**Z value**	**OR**	**P Value**	**β**	**Z value**	**OR**	**P Value**
	**(SE)**		**(95% CI)**		**(SE)**		**(95% CI)**	
Age	0.03	0.75	1.03	0.452	0.05	1.00	1.05	0.317
	(0.04)		(0.95,1.12)		(0.05)		(0.95,1.16)	
Sex	-1.98	-2.33	0.14	0.020	-2.45	-2.40	0.09	0.016
(Female vs. Male)	(0.85)		(0.03,0.71)		(1.02)		(0.01,0.62)	
BMI	-0.10	-1.25	0.91	0.211	-0.12	-1.33	0.89	0.183
	(0.08)		(0.78,1.06)		(0.09)		(0.75,1.05)	
Surgical approach	0.85	1.42	2.34	0.156	1.02	1.57	2.77	0.117
	(0.60)		(0.72,7.60)		(0.65)		(0.77,9.96)	
Follow-up duration	0.18	2.57	1.20	0.010	0.15	2.50	1.16	0.012
	(0.07)		(1.04,1.38)		(0.06)		(1.03,1.31)	
Preoperative MCS	0.82	6.83	2.27	<0.001	0.78	7.80	2.18	<0.001
	(0.12)		(1.78,2.90)		(0.10)		(1.80,2.65)	

Bolded *P* values indicate statistical significance (*P* < 0.05)

*OR* Odds ratio, *CI* Confidence interval, *SE* Standard error

## Discussion

Depression and anxiety are prevalent mental health conditions that significantly impact postoperative pain relief and health-related quality of life, particularly for patients undergoing spinal surgery [10]. In China, the lifetime prevalence of these disorders is notable, and it is well-established that their incidence among patients with degenerative spinal diseases is two to three times higher than in the general population [11, 12]. Approximately one-third of patients scheduled for spinal surgery experience these adverse psychological states preoperatively, which are known to be strong predictors of poorer patient-reported outcomes (PROs) and diminished rehabilitation effectiveness [13, 14].

Most current studies indicate that preoperative psychological distress predicts poorer surgical outcomes [15, 16]. For instance, research on lumbar fusion populations, such as the studies by Anderson [17] et al. and Miller [18] et al., has demonstrated a significant correlation between depression and suboptimal surgical efficacy, poor postoperative recovery, and decreased patient satisfaction. This correlation is often attributed to mechanisms such as an exacerbated perception of pain [19], reduced motivation for rehabilitation [20], and potential alterations in pain processing pathways [21–23]. Similarly, studies focusing on cervical surgery, includeing those by Alvin [24] et al. and Oshima [25] et al., have reported that preoperative depression and anxiety are associated with intensified postoperative pain, poorer functional gains, and worse overall quality of life.

Against this backdrop, the results of our study present a clinical contradiction. Despite a significant proportion (approximately 31%) of the CSM cohort presenting with preoperative psychological distress, their functional and physical health outcomes at final follow-up demonstrated no statistically significant divergence from those of patients without such distress. Crucially, both groups showed comparable improvements in the NDI and the physical component of the SF-12 survey. This equivalence in therapeutic results, which contradicts the prevailing literature, necessitates a thorough investigation into the underlying mechanisms that facilitated this unexpected convergence in outcomes.

Two primary, interconnected factors are hypothesized to underlie this discrepancy. The first involves the inherently subjective nature of pain and disability assessment. Pain transcends mere sensory input; it is a multifaceted experience profoundly influenced by emotional and cognitive dimensions. Consequently, standardized metrics such as the VAS, Numeric Rating Scale (NRS), and NDI are deeply contingent on a patient’s subjective self-report [26]. Individuals with preoperative psychological distress frequently present with concomitant emotional disorders, which can introduce a significant cognitive-affective bias. This may manifest preoperatively as a heightened perception of pain and functional limitation, thereby inflating baseline scores. This relationship is underpinned by a well-documented pathophysiological link: chronic pain and affective disorders such as depression and anxiety share common neural pathways. They engage in a vicious, self-perpetuating cycle wherein pain exacerbates low mood, and the resulting psychological distress, in turn, amplifies the perception of pain by effectively lowering the pain threshold.

However, following surgery, these patients experience a genuine and substantial objective improvement. The study noted that the JOA scores in the psychological distress group improved by over 60%, indicating a significant objective enhancement in neurological function and mobility due to the alleviation of spinal cord compression. This objective improvement likely mitigates the subjective cognitive bias that inflated their preoperative scores. As nerve function and muscle strength recover, the primary driver of the pain-depression cycle is broken. The drastic reduction in objective disability allows for a recalibration of the patient’s subjective experience, enabling their reported outcomes to converge with those of patients who started from a less psychologically influenced baseline.

The second factor concerns the distinct nature of CSM. Unlike pain-based conditions, CSM causes objective neurological impairments like gait instability and motor weakness [27]. This profound disability is a direct cause of depression and anxiety, meaning preoperative distress may be a reaction to the disease rather than a pre-existing condition.

Surgery for CSM directly targets this root cause by decompressing nerves and restoring function. This objective recovery—distinct from subjective pain relief—directly mitigates the helplessness and dependence that fuel psychological distress. The tangible improvement in mobility and independence creates powerful positive feedback for mental well-being.

This is evidenced by the marked postoperative improvement in MCS scores. Nearly half of the distressed patients achieved normative mental health scores, demonstrating that resolving the physical pathology can concurrently alleviate the secondary psychological distress it caused.

Furthermore, as time progresses post-surgery, patients gain a deeper understanding of their condition and recovery trajectory, which reduces the “fear of the unknown” that fuels anxiety. Apaydin [5, 20, 28]findings revealed a significant negative correlation between the NDI scores of female patients and their quality of life, Furthermore, the researchers identified age as a significant influencing factor within this demographic. These factors exacerbate anxiety and depression, further detrimentally impacting the overall quality of life of these patients. The study also hints at demographic factors, such as the role of age and gender in influencing quality of life perceptions, noting that female patients often report lower baseline scores due to a complex interplay of biomechanical and sociocultural factors. Yet, these too showed improvement over time, suggesting that the objective benefits of surgery eventually outweigh these initial influencing factors.

In conclusion, patients with CSM frequently experience high levels of psychological distress. Anterior surgical intervention can lead to significant improvements in disease-related scores and overall quality of life. Notably, among female patients, low preoperative MCS and shorter follow-up periods are key factors influencing the lack of improvement in postoperative MCS. Importantly, however, preoperative psychological distress does not adversely affect the enhancement of neurological function or overall satisfaction following surgery.

Limitations of this study: (1) This investigation was designed as a single-center retrospective analysis with a relatively small sample size. Multicenter studies are needed to generalize the results. (2) The chosen follow-up time points—preoperative and at the final assessment—result in a significant time interval, which may fail to accurately reflect the immediate psychological changes experienced by patients following surgery. (3) The surgical procedures were carried out by different surgical teams, which may introduce variability in operative techniques and communication regarding the patient’s condition, potentially influencing the outcomes of the study. (4) Patients diagnosed with CSM were not categorized according to the severity of their neurological impairment, thereby introducing the possibility of bias in the results, and without comparing to a non-surgical group. (5) Relying solely on the SF-12 MCS subitem scores may fail to cover the entire spectrum of emotional disorders, which is a limitation in assessing psychological distress.

## Data Availability

The data generated and analyzed during this study are available from the corresponding author upon reasonable request.

## Abbreviations

ACCF: Anterior cervical corpectomy and fusion
ACDF: Anterior cervical discectomy and fusion
ADF: Anterior and decompression and fusion
mJOA: modified Japanese Orthopaedic Association
NDI: Neck disability index
MCS: Mental component score
PCS: Physical component score
